# Identification of Dutch hospital inpatients with possible palliative care needs: a nation-wide flash mob study

**DOI:** 10.1186/s12904-026-02008-0

**Published:** 2026-02-13

**Authors:** Annette WG van der Velden, Albert H de Heij, Marieke HJ van den Beuken, Evelien JM Kuip, Ginette M Hesselmann, Ellen JM de Nijs, Bregje AA Huisman, Astrid W Oosten, Anna KL Reyners, Pauline de Graeff

**Affiliations:** 1 University Medical Center Groningen, University of Groningen, Expertise Centre Palliative Care, Hanzeplein 1, Groningen, 9713GZ the Netherlands; 2 Maastricht University Medical Center, Expertise Centre Palliative Care, Maastricht, the Netherlands; 3 Radboud University Medical Center, Expertise Centre Palliative Care, Nijmegen, the Netherlands; 4 University Medical Center Utrecht, Expertise Centre Palliative Care, Utrecht, the Netherlands; 5 Leids University Medical Center, Expertise Centre Palliative Care, Leiden, the Netherlands; 6 Amsterdam University Medical Centers, Expertise Centre Palliative Care, Amsterdam, the Netherlands; 7 Erasmus Medical Center, Expertise Centre Palliative Care, Rotterdam, the Netherlands

**Keywords:** Surprise question, Palliative care, Life-limiting condition, Palliative care team involvement, Cross-sectional study.

## Abstract

**Background:**

Identifying hospital inpatients who are approaching the end of life is essential for providing optimal palliative care. The study aims to determine the prevalence of hospital inpatients with a potentially limited life expectancy (< 12 months) using the surprise question, and to assess the extent of specialist palliative care team (PCT) involvement in this population.

**Methods:**

On the 16^th^ of April 2021, we conducted a multi-center, cross-sectional, nationwide study in Dutch hospitals using a flash mob design. Nurses and physicians independently answered the surprise question (SQ) for all adult hospital inpatients: “Would you be surprised if this patient died within the next 12 months?”. If answered negatively, they were then asked to indicate if they estimated life expectancy to be less than 3 months, and whether PCTs were involved.

**Results:**

In total, 48 of 68 (70%) Dutch hospitals participated in the study. Nurses and physicians filled out 16,607 questionnaires regarding 8,768 patients.

Of 8,768 patients, 80% were admitted because of non-malignant diseases. Physicians answered the SQ negatively for 2,826 (35%) patients. Physicians indicated that PCT was involved in 6% of these patients and that PCT referral was considered useful for another 6%. For patients with an estimated life expectancy < 3 months (n=582), these proportions increased to 15% and 13%, respectively.

**Conclusions:**

A third of all hospital inpatients had a possible life-limiting condition and may benefit from palliative care and advance care planning. Future studies should focus on exploring the palliative care needs of hospital inpatients and possible benefits of timely implementation of palliative care.

**Supplementary Information:**

The online version contains supplementary material available at 10.1186/s12904-026-02008-0.

## Background

When faced with a life-limiting condition, most people prefer to die at home [1]. However, in Europe, 22–66% of patients are admitted to the hospital during their last year of life and 26–68% of patients die in this setting [2, 3]. In the Netherlands, 46% of deceased patients were admitted to the hospital in the last 3 months of life and up to 60% in the last 12 months [4, 5]. No data is available on whether those patients were timely identified as having a limited life expectancy, and whether appropriate palliative care was provided.

The purpose of palliative care is to improve the quality of life of patients and their families who are facing problems associated with a life-limiting condition. It prevents and relieves suffering due to early identification and adequate assessment and treatment of pain and other problems, whether physical, psychosocial or spiritual [6]. In the Netherlands, unlike many other countries, palliative care is defined as generalist care that can be offered by all healthcare providers at home, in nursing homes, hospices, and hospitals. However, specialized palliative care professionals can provide additional support in all care settings.

To provide optimal generalist and specialist palliative care, knowing which hospital inpatients may be approaching the end of life is essential. The study [3] cited above was conducted nationwide in all public Danish hospitals and used prospective record linkage data. A 2011 study [7] in 14 Belgian hospitals showed that 9.5% of the hospital inpatients had an incurable, progressive, life-limiting condition with no possibility of improvement. Research in several other countries revealed that the proportion of inpatients needing palliative care ranges between 13% and 41% [8–11]. In the Netherlands, however, the proportion of hospital inpatients needing palliative care due to a potentially life-limiting condition is unknown. In addition, scarce data on the involvement of specialist palliative care teams (PCT) is available. However, surveys show that specialist palliative care teams’ involvement increased from 0.9 to 1.7% of all patient admissions in Dutch hospitals between 2017 and 2024 [12].

The study aims to determine the prevalence of hospital inpatients with a potentially limited life expectancy (< 12 months) using the surprise question, and to assess the extent of specialist palliative care team (PCT) involvement in this population.

## Methods

### Study design, setting, and participants

This nationwide, single-day, multicenter, cross-sectional, observational study was carried out in the form of a “flash mob study” [13]. The flash mob design enables a large group of researchers to rapidly collect data in a short time frame to answer a relatively straightforward question. For this flash mob study, all data were collected at the participating hospitals in the Netherlands on a single day: the 16th of April 2021.

All hospitals in the Netherlands (*n* = 68) were invited by e-mail and telephone to participate in this study. At each participating hospital, a local study coordinator was responsible for study procedures. The local study coordinator received training through a webinar in which the study procedures were explained and practical tips and tricks were shared. In addition, all instructions were provided to them in written form. Within each hospital, these materials could be utilized in a manner tailored to the needs of the participating staff members.

All patients at the participating hospitals who were admitted on the 16th of April 2021 were eligible for inclusion except for those admitted to the emergency department (excluded because of rapidly changing and brief patient contacts), the day treatment center and the obstetric and pediatric wards. Each local study coordinator invited the medical specialties to participate in this study. Data was anonymized. Therefore, according to general data protection regulations, informed consent from hospitalized patients was not required. The study was assessed by the medical ethical committee of the UMCG and by all participating hospitals (Trial NL9184).

### Measurements

All hospital beds at the participating wards in the included hospitals were allocated a study number which could be digitally entered into the data management system REDCap (Supplement 1). For occupied beds, the questionnaire was filled out by the responsible nurses and physicians (medical specialists, residents, nurse practitioners, or physician assistants). Beds not occupied by patients were also registered to ensure a complete overview of admitted patients relative to the maximum capacity. To identify patients with a potentially life-limiting condition who could benefit from palliative care, we used the surprise question (SQ) as an assessment tool. For all included patients, nurses and physicians independently answered the surprise question, “Would you be surprised if this patient died within the next 12 months?” [14]. If the respondents gave a negative answer to the SQ, we asked the follow-up question, “Is the estimated life expectancy less than 3 months?” All nurses and physicians were then asked about current involvement of the PCT, or whether this would be useful. In addition, nurses were asked to fill out a minimal data set of categorized patient characteristics: age (< 70 years or ≥ 70 years), gender, diagnosis at admission (malignant, non-malignant, or COVID-19 (suspected or confirmed), medical specialty (a complete list is provided in Supplement 2) and documented treatment limitations (positive if any treatment limitation was recorded regarding cardiopulmonary resuscitation, mechanical ventilation and/or ICU admission).

### Statistical analysis

The proportion of hospital inpatients with a possible life-limiting condition was estimated by a negatively answered SQ.

To analyze the interobserver agreement between nurses and physicians Cohen’s Kappa coefficient [15] was used, and interpreted as follows: 0.01–0.20 slight agreement; 0.21– 0.40 fair; 0.41–0.60 moderate; 0.61–0.80 substantial and 0.81–0.99 almost perfect agreement.

To investigate the association between the answer to the SQ and patient or hospital characteristics, chi-square tests were performed using categorized variables.

Participating hospitals were categorized into academic and non-academic hospitals. The medical specialties were classified as either non-surgical or surgical (a full description is provided in Supplement 3). Medical specialties with less than ten admitted patients were excluded from the analysis. A p-value of < 0.05 was considered statistically significant.

Statistical analyses were performed using SPSS version 23 (IBM SPSS Statistics, IBM Corporation).

## Results

### Participants

**Fig. 1:**
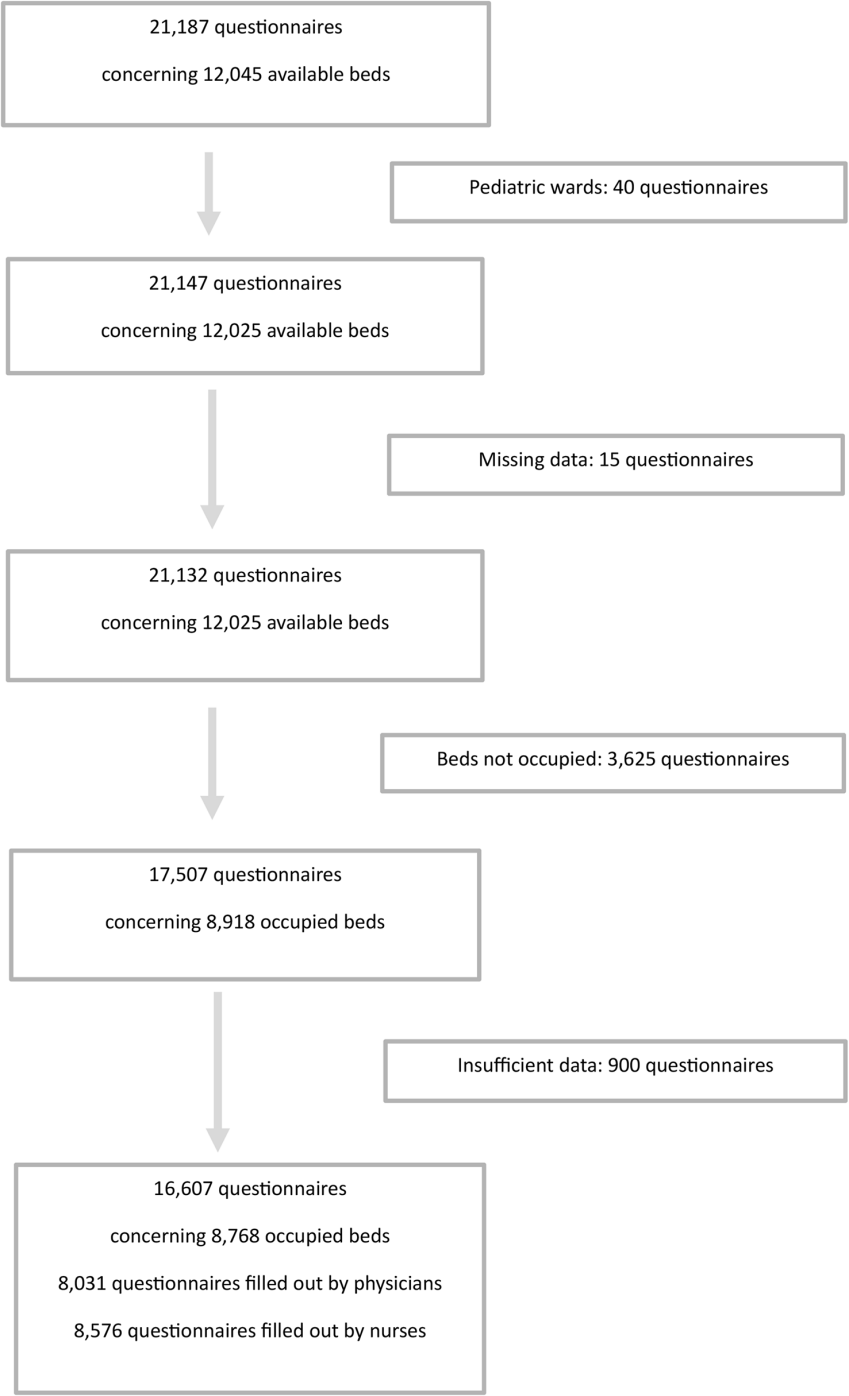
Flow chart of questionnaires

Forty-eight out of 68 (70%) Dutch hospitals participated in the study. Among the 48 hospitals, all 8 university hospitals were included, together with 1 specialized oncological hospital and 39 general hospitals. The participating hospitals were evenly distributed across the Dutch provinces, thereby ensuring that this study provided a representative overview of the current situation in the Netherlands. At most participating hospitals, the majority of specialties participated. However, intensive care and psychiatric specialties participated at around 30% of the hospitals. The number of participating patients per specialty, and a complete list of participating medical specialties is provided in Supplement 3. The numbers of participating medical specialties in the hospitals and the proportion (%) relative to all specialties at each hospital is shown in Supplement 4. A total of 16,607 questionnaires concerning 8,768 patients could be used for analyses, of which nurses filled out 8,576 and physicians 8,031 (Fig. 1). For 7,803 patients, the SQ was answered by both the nurse and physician.

### Descriptive data

Overall, a slight majority of patients (53%) were younger than 70 years of age. A large majority (80%) of patients were admitted due to non-malignant diseases, including 12.5% with suspected or confirmed COVID-19. Two-thirds (67%) of patients were admitted for a non-surgical specialty (Table 1).

**Table 1:** Baseline characteristics of the study population

	*n* (%)
Hospital	
Academic hospitals	2178 (24.8)
Non-academic hospitals	6590 (75.2)
Gender	
Male	4600 (53.9)
Female	3927 (46.1)
Reason for admission	
Malignant disease	1693 (19.9)
Non-malignant disease	5767 (67.6)
(Suspected) COVID-19	1067 (12.5)
Medical specialty	
Non-surgical specialty	5707 (66.9)
Surgical specialty	2791 (32.7)
Other^*^	28 (0.3)
Recorded treatment limitations	
Yes	3327 (39.0)
No	5199 (61.0)

Data on age, gender and reason for admission: *n*=8527; data on medical specialty and treatment limitations: *n*=8526; data on type of hospital: *n*=8768

* Pain medicine, transplantation medicine, trial ward or acute admission ward

### Main results

**Fig. 2:**
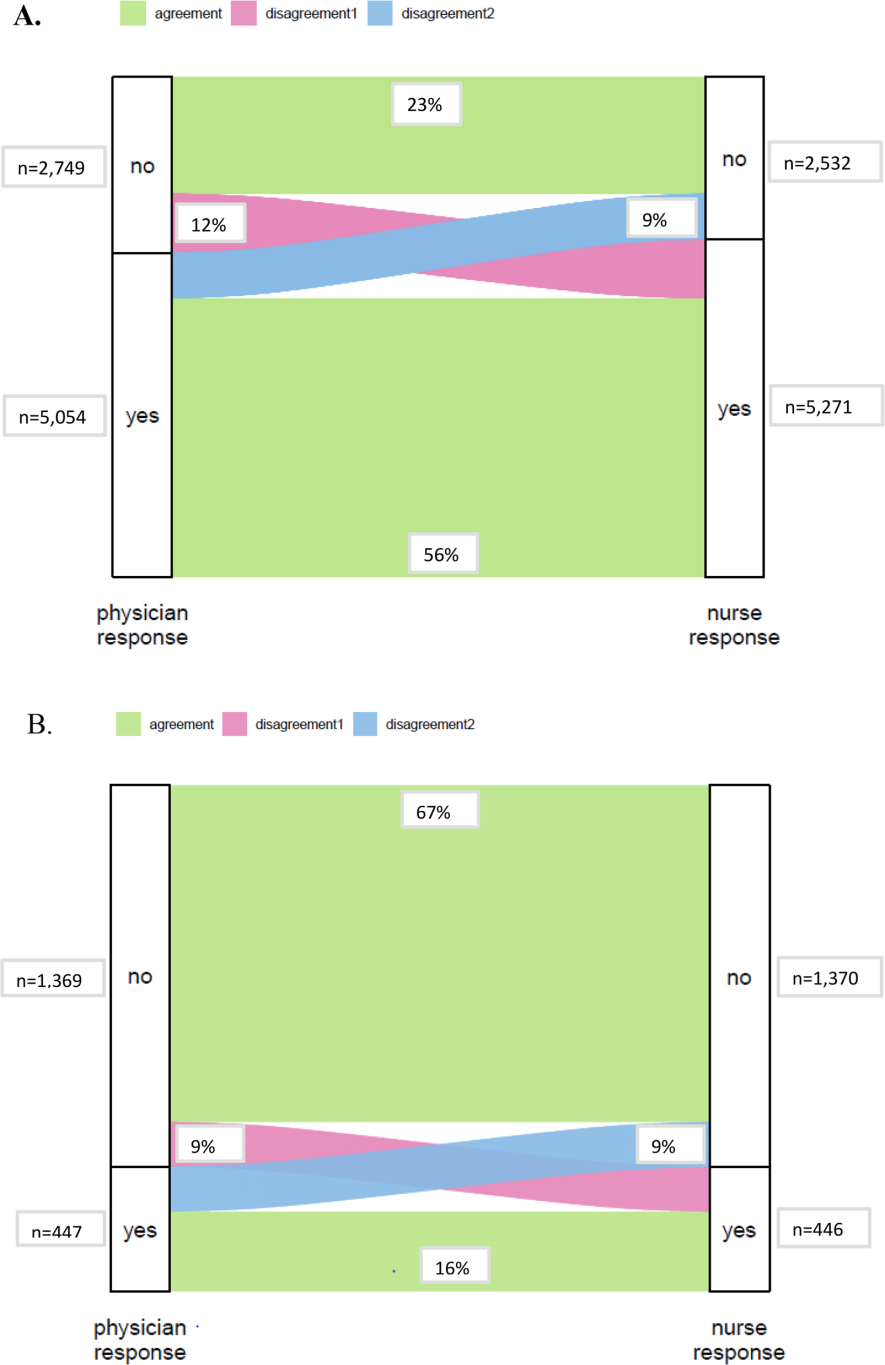
Alluvial plots for individual agreement/disagreement about estimated life expectancy of the same patient by nurses and physicians. Disagreement 1: the physician answered no and the nurse yes; Disagreement 2: the nurse answered no and the physician yes. **A** Surprise Question: “Would you be surprised if this patient died within the next 12 months?” (*n*=7,803). **B** Expected life expectancy less than 3 months (*n*=1,816)

The SQ was answered negatively by nurses for 2,745 patients (32%) and by physicians for 2,826 (35%) patients (Fig. 2). The concordance between nurses and physicians was moderate, with a kappa of 0.53. Disagreement between nurses’ and physicians’ answers to the SQ was found in 21% of patients (Fig. 2). In the total population, life expectancy was estimated to be less than 3 months for 583 patients (7%) by nurses and 582 patients (7%) by physicians. In this subgroup, the agreement between nurses and physicians was moderate (kappa 0.52), and disagreement was found in 18% of patients. A post hoc analysis was conducted in patients with discordant responses to the surprise question between nurses and physicians (*n* = 1643). This analysis demonstrated significantly greater discordance among patients aged ≥ 70 years (16.8% < 70 years vs. 25.8% ≥ 70 years) and among those admitted with malignant disease compared to non-malignant disease/COVID-19 (25.4% malignant disease vs. 19.9% and 21% non-malignant disease/COVID-19, respectively). At the same time, no difference was observed between patients admitted to surgical versus medical departments.

Table 2 shows the patient characteristics and involvement of the PCT classified by the SQ response. The SQ was answered negatively significantly more often in patients 70 years or older compared to younger patients, patients admitted with a diagnosis of malignant disease compared to patients with a diagnosis of non-malignant disease, and patients admitted for non-surgical specialties compared to surgical specialties. Treatment limitations were not recorded for 1,046 (38%) patients when the SQ was answered negatively and for 889 (41%) patients with a life expectancy of less than 3 months. For patients with a negatively answered SQ by physicians, a specialist PCT was involved in 151 (6%) patients and was considered useful for 157 (6%). For the subgroup of patients with a negative SQ and a life expectancy of less than 3 months, a specialist PCT was involved in 87 (15%) of patients and was considered useful for 75 (13%).

**Table 2:** Subgroup analysis

	Nurse			Physician		
	SQ no *n* (%)	SQ yes *n* (%)	*p*-value	SQ no *n* (%)	SQ yes *n* (%)	*p*-value
Age						
< 70 years	978 (21.6)	3549 (78.4)	< 0.001	922 (22.4)	3190 (77.6)	< 0.001
≥ 70 years	1767 (44.2)	2227 (55.8)		1828 (49.5)	1867 (50.5)	
Gender						
Male	1482 (32.2)	3114 (67.8)	0.947	1459 (34.7)	2742 (65.3)	0.323
Female	1263 (32.2)	2662 (67.8)		1291 (35.8)	2315 (64.2)	
Reason for admission						
Malignant disease	786 (46.5)	903 (53.5)	< 0.001	709 (48.5)	752 (51.5)	< 0.001
Non-malignant disease	1634 (28.3)	4131 (71.7)		1723 (32.0)	3660 (68.0)	
(Suspected) COVID-19	325 (30.5)	742 (69.5)		318 (33.0)	645 (67.0)	
Medical specialty						
Non-surgical specialty	2121 (37.2)	3582 (62.8)	< 0.001	2149 (41.1)	3086 (58.9)	< 0.001
Surgical specialty	621 (22.3)	2168 (77.7)		595 (23.4)	1950 (76.6)	
Other *	3 (10.7)	25 (89.3)		6 (21.4)	22 (78.6)	
Documented treatment limitations						
Yes	1756 (64.0)	1571 (27.2)	< 0.001	1704 (62.0)	1364 (27.0)	< 0.001
No	989 (36.0)	4205 (72.8)		1046 (38.0)	3692 (73.0)	
Life expectancy						
< 3 months	583 (21.3)	NA	< 0.001	582 (20.6)	NA	< 0.001
3–12 months	2155 (78.7)			2241 (79.4)		
Involvement of PCT						
Yes	166 (6.0)	29 (0.5)	< 0.001	157 (5.6)	17 (0.3)	< 0.001
No	2396 (87.3)	5732 (99.2)		2510 (88.8)	5150 (99.5)	
Desired	183 (6.7)	15 (0.3)		157 (5.6)	11 (0.2)	

The responses to the Surprise Question (SQ) by nurses and physicians for age, gender, reason for admission, medical specialty, documented treatment limitations, life expectancy and involvement of palliative care team (PCT)

*NA* Not Applicable, *PCT* Palliative Care Team, *SQ* Surprise Question

*  Pain medicine, transplantation medicine, study ward or acute admission ward

The responses to the Surprise Question (SQ) by nurses and physicians for age, gender, reason for admission, medical specialty, documented treatment limitations, life expectancy and involvement of palliative care team (PCT).

**Fig. 3:**
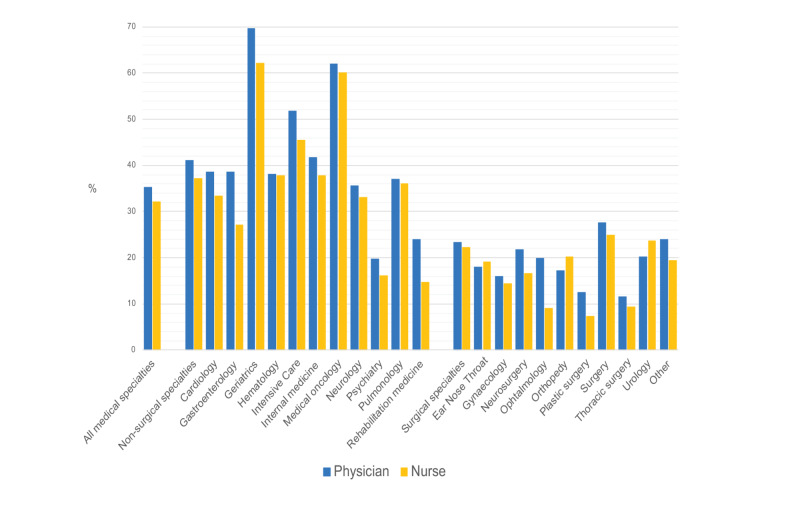
Surprise question negatively answered according to hospitalization medical specialties (%)

Figure 3 shows the differences between specialties regarding patients with a negative SQ. Most patients with an SQ answered negatively by physicians were admitted for a geriatric (70%) or medical oncology specialty (62%). For patients admitted for a non-surgical specialty, physicians answered the SQ negatively (42%) more often than those for surgical specialties (19%).

## Discussion

This nationwide flash mob study showed that about one-third of hospital admissions in the Netherlands involved care for patients with a potentially life-limiting condition. These patients may benefit from palliative care and advance care planning. Specialist PCTs were involved in the care for only 6% of all patients for whom the SQ was answered negatively, and their involvement was considered useful for another 6%. For the subgroup with an estimated life expectancy of less than 3 months, specialist PCTs were involved in 15% of cases, and their involvement was considered useful for another 13%. Treatment limitations were not recorded for 41% of patients with a life expectancy of less than 3 months.

This study was the first to provide a nationwide estimate of the proportion of hospital inpatients with a possibly limited life expectancy using the SQ by medical and nursing staff. Previous attempts have been made to clarify the number of hospital admissions of patients in their last year of life, predominantly by the retrospective use of patient records and death registries. Jarlbaek et al. [3], for example, used this method in Denmark and found that 22% of the inpatients died within a year, especially older patients and those who were admitted for a more extended period. Of the deceased patients, 50% were admitted to oncological and hematological wards and 15% to surgical wards. This data is comparable to our findings, where oncological and surgical specialists/nurses answered the SQ negatively in 62% and 19% of cases, respectively.

Overbeek et al. studied admissions of patients to care facilities in their last year of life in 15 European countries and Israel [2]. They found that the proportion of such patients ranged from 54% in France to 76% in Austria, Israel, and Slovenia. Although these admissions mainly concerned hospitalizations, they also included admissions to hospices and nursing homes. The main difference with our flash mob study was that Overbeek et al. included only community-dwelling older people. Moreover, in that study, healthcare providers were not asked to assess possible palliative care needs of patients. An important finding of this study is that 80% of inpatients identified as having a potentially limited life expectancy were admitted with non-malignant conditions. Increasing awareness among hospital clinicians that patients with advanced non-malignant conditions—such as chronic obstructive pulmonary disease (COPD), heart failure, end-stage renal failure (ESRF) and neurodegenerative disease may benefit from timely palliative care is essential. Prognostic uncertainty in these populations should not delay referral, as palliative care can support symptom control, advance care planning, and shared decision-making regardless of diagnosis. Interventions aimed at improving education, implementing validated identification tools systematically, and normalizing palliative care involvement for non-malignant disease trajectories may help in access to palliative care services.This study showed limited involvement of PCTs in Dutch hospital inpatients. In contrast, results of a previous study performed in New South Wales that described the healthcare utilization of patients during their final year of life showed that 15% of patients were seen by a specialist PCT [16]. In the Netherlands, palliative care is considered a core responsibility of hospital physicians, nurse practitioners, general practitioners and community nurses, with specialist palliative care teams functioning primarily as consultative services rather than routine providers of care. This organisational model assumes that most palliative needs can be met by generalist clinicians with specialist input required only for complex situations. The limited involvement of specialist Palliative Care Teams among Dutch hospital inpatients reflects the strong reliance on generalist healthcare professionals. This generalist-specialist model was confirmed in a study by Boddaert et al. [17], who evaluated the quality of life among Dutch cancer patients. In that study, 76% of patients received palliative care in their final year before death. Of this group, only 12% received care provided by palliative care specialists. Optimizing the balance between generalist and specialist palliative care provision will be essential to improve patient outcomes and ensure that healthcare resources are deployed effectively. Difficulties in recognizing patients who may benefit from specialist involvement combined with limited awareness of referral options highlight potential gaps in clinical pathways and national guidelines. Given the evidence that earlier palliative care initiation is associated with reduced potentially inappropriate end-of-life care [17], greater policy emphasis on early identification and clearer referral criteria may be warranted. Measures to encourage a more timely start of palliative care include combining the SQ with treatment limitations as part of grand rounds and electronic medical records, or even earlier by focusing on palliative care and advance care planning at home before admission. The findings of this study also underscore the need for enhanced educational strategies to strengthen generalist palliative care competencies, alongside research to better understand barriers to referral and to evaluate interventions that facilitate timely specialist input. Strength and limitations.

The study had notable strengths due to the flash mob design. Using this design, we could answer a clinically relevant question quickly. The simplicity of the design facilitated the participation of most Dutch hospitals and involved healthcare providers who often need to be more engaged in clinical research projects. It also resulted in a large sample size, required little investment of time and resources from the participating hospitals, and probably entailed slight inclusion bias. The influence of the COVID-19 pandemic on the study was relatively minor. Although the pandemic initially delayed the study, the final number of participating hospitals was not affected. Nevertheless, several individual wards were unable to participate due to pandemic-related circumstances. Several other limitations should also be mentioned. First, due to the flash mob design, we were only able to collect a limited data set. Subsequent studies could benefit from a more comprehensive analysis, incorporating variables such as frailty status and documentation of advance care planning. Second, the cross-sectional nature of the design meant that we had no data on the actual lifespans of the patients and had no reports on possible discussions about future care plans at or after discharge. Third, although previous research has shown that patients approaching the end of life appreciate prompt, personalized symptom management, holistic support, guidance in decision-making, and preparation for the future in consultations with specialist PCTs [18], we did not ask the patients and their relatives about their desire for palliative care. Finally, the SQ as a predictive tool for identifying patients with a limited life expectancy has shown wide variation in sensitivity and specificity [19].However, due to the absence of better alternatives, determining the estimated life expectancy of hospital inpatients with the SQ is the most practical choice. The Dutch palliative care framework uses the SQ to create awareness of possible limited life expectancy [20]. The SQ provides information that may influence decisions regarding meaningful healthcare interventions. Additionally, it should encourage healthcare providers to engage in conversations with their patients regarding their wishes and goals for their care.

## Conclusions

This flash mob study showed that one-third of the patients admitted to Dutch hospitals will likely be in their last 12 months of life. This outcome underlines the importance of raising awareness of the palliative care needs in hospital inpatients. More research (qualitative and quantitative) is needed to explore the reasons for the limited involvement of specialized hospital palliative care teams and the need for general and specialized palliative care among hospital inpatients in a mixed generalist and specialist palliative care model.

## Supplementary Information

Supplementary Material 1: Supplement 1. Clinical Research Form (CRF) of the survey in REDCap. Supplement 2. Classification of medical specialties. Supplement 3. Number and proportion (%) of participating patients of medical specialties. Supplement 4. Number of participating medical specialties in the hospitals and proportion (%) relative to all medical specialties.

## Data Availability

All data generated or analyzed during this study are included in this published article and its supplementary information files. The dataset is available from the corresponding author on reasonable request.

## Abbreviations

COVID-19: Coronavirus Disease of 2019
HCP: Healthcare Professional
ICU: Intensive Care Unit
PCT: palliative care teams
SQ: surprise question
UMCG: University Medical Center Groningen
