# Supplementary material for: Identification of Dutch hospital inpatients with possible palliative care needs: a nation-wide flash mob study
Source: BMC Palliat Care. 2026 Feb 13;25:69. doi: 10.1186/s12904-026-02008-0 (PMC13005480; doi:10.1186/s12904-026-02008-0)
Supplement: Supplementary file 1 — Supplementary Material 1: Supplement 1. Clinical Research Form (CRF) of the survey in REDCap. Supplement 2. Classification of medical specialties. Supplement 3. Number and proportion (%) of participating patients of medical specialties. Supplement 4. Number of participating medical specialties in the hospitals and proportion (%) relative to all medical specialties. [file 12904_2026_2008_MOESM1_ESM.docx]

**Additional files**

- Additional file 1. Clinical Research Form (CRF) of the survey in REDCap
- Additional file 2. Classification of medical specialties
- Additional file 3. Number and proportion (%) of participating patients of medical specialties
- Additional file 4. Number of participating medical specialties in the hospitals and proportion (%) relative to all medical specialties

**Additional file 1. Clinical Research Form (CRF) of the survey in REDCap**

General part

Registration number:

Bed occupied:

- Yes
- No

Data importer:

- Physician/physician assistant/nurse specialist
- Nurse

Questionnaire for nurse

Registration number:

1. Hospitalization medical specialty

- Cardiology
- Surgery
- Dermatology
- Geriatrics
- Gynecology
- Hematology
- Intensive care
- Internal medicine
- Medical oncology
- Ear Nose Throat
- Pulmonology
- Gastroenterology
- Neurology
- Neurosurgery
- Ophthalmology
- Orthopedics
- Plastic surgery
- Psychiatrics
- Thoracic surgery
- Urology
- Other:

1. Age:

- < 70 years
- ≥70 years

1. Gender:

- Male
- Female

1. Reason for admission:

- Malignant disease
- Non-malignant disease
- Suspected/confirmed COVID-19

1. Are treatment limitations documented?

- Yes
- No

1. Was the palliative care team involved during this admission or in last month?

- Yes
- No
- Not yet, but this is desired

1. Would I be surprised if this patient died within the next 12 months?

- Yes, end of questionnaire
- No, go to question 8

1. If the answer is no for question 7, is the expected life expectancy less than 3 months?

- Yes
- No

Questionnaire for physician/physician assistant/nurse specialist

Registration number:

1. Would I be surprised if this patient died within the next 12 months?

- Yes, end of questionnaire
- No, go to question 3

1. If the answer is no for question 1, is the expected life expectancy less than 3 months?

- Yes
- No

1. Was the palliative care team involved during this admission or in last month?

- Yes
- No
- Not yet, but this is desired

**Additional file 2. Classification of medical specialties**

| Non-surgical specialties | Surgical specialties |
| --- | --- |
| Cardiology | Ear Nose Throat |
| Gastroenterology | Gynecology |
| Geriatrics | Neurosurgery |
| Hematology | Ophthalmology |
| Intensive care | Orthopedics |
| Internal medicine | Plastic surgery |
| Medical oncology | Surgery |
| Neurology | Thoracic surgery |
| Psychiatrics | Urology |
| Pulmonology | Other^*^ |
| Rehabilitation medicine |  |

* Pain medicine , transplantation medicine , study ward or acute admission ward

**Additional file 3. Number and proportion (%) of participating patients of medical specialties**

|  | n (%) |
| --- | --- |
| Cardiology | 1087 (12.7) |
| Surgery | 1575 (18.5) |
| Geriatrics | 246 (2.9) |
| Gynecology | 110 (1.3) |
| Hematology | 256 (3.0) |
| Internal medicine | 1181 (13.9) |
| Pulmonology | 1091 (12.8) |
| Gastroenterology | 398 (4.7) |
| Neurology | 723 (8.5) |
| Neurosurgery | 151 (1.8) |
| Plastic surgery | 27 (0.3) |
| Psychiatrics | 93 (1.1) |
| Thoracic surgery | 117 (1.4) |
| Urology | 291 (3.4) |
| Orthopedics | 452 (5.3) |
| Intensive care | 156 (1.8) |
| Ear Nose Throat | 68 (0.8) |
| Ophthalmology | 11 (0.1) |
| Other^*^ | 77 (0.9) |
| Medical oncology | 353 (4.1) |
| Rehabilitation medicine | 54 (0.6) |

* Pain medicine , transplantation medicine , study ward or acute admission ward

**Additional file 4. Number of participating medical specialties in the hospitals and proportion (%) relative to all medical specialties**

|  | n (%) |
| --- | --- |
| Cardiology | 47 out of 47 (100) |
| Surgery | 46 out of 48 (96) |
| Geriatrics | 46 out of 47 (98) |
| Gynecology | 36 out of 47 (77) |
| Hematology | 46 out of 47 (98) |
| Internal medicine | 48 out of 48 (100) |
| Pulmonology | 48 out of 48 (100) |
| Gastroenterology | 47 out of 48 (98) |
| Neurology | 45 out of 47 (96) |
| Neurosurgery | 38 out of 40 (95) |
| Plastic surgery | 30 out of 36 (83) |
| Psychiatrics | 9 out of 25 (36) |
| Thoracic surgery | 19 out of 22 (86) |
| Urology | 45 out of 48 (94) |
| Orthopedics | 43 out of 45 (96) |
| Intensive care | 14 out of 48 (29) |
| Ear Nose Throat | 16 out of 18 (89) |
| Ophthalmology | 9 out of 10 (90) |
| Medical oncology | 48 out of 48 (100) |
